# Anti-Xa Activity of Enoxaparin for Prevention of Venous Thromboembolism in Severe Nephrotic Syndrome—A Single Center Prospective Study

**DOI:** 10.3390/jcm10235709

**Published:** 2021-12-06

**Authors:** Anna Matyjek, Aleksandra Rymarz, Zuzanna Nowicka, Slawomir Literacki, Tomasz Rozmyslowicz, Stanislaw Niemczyk

**Affiliations:** 1 Department of Internal Diseases, Nephrology and Dialysis, Military Institute of Medicine, 04-141 Warsaw, Poland; arymarz@wim.mil.pl (A.R.); sniemczyk@wim.mil.pl (S.N.); 2 Department of Biostatistics and Translational Medicine, Medical University of Lodz, 92-215 Lodz, Poland; zuzanna.nowicka@umed.lodz.pl; 3 Department of Laboratory Diagnostics, Military Institute of Medicine, 04-141 Warsaw, Poland; sliteracki@wim.mil.pl; 4 Department of Pathology and Laboratory Medicine, University of Pennsylvania, Philadelphia, PA 19104, USA; rozmyslo@pennmedicine.upenn.edu

**Keywords:** antifactor Xa, anti-Xa activity, enoxaparin, low-molecular-weight heparin, nephrotic syndrome, thromboembolism

## Abstract

Severe nephrotic syndrome (NS) is associated with high risk of venous thromboembolic events (VTE), as well as presumably altered heparin pharmacokinetics and pharmacodynamics. Although prophylactic anticoagulation is recommended, the optimal dose is not established. The aim of the study was to test two co-primary hypotheses: of reduced enoxaparin effectiveness and of the need for dose-adjustment in NS. Forty two nephrotic patients with serum albumin ≤2.5 g/dL were alternately assigned to a standard fixed-dose of enoxaparin (NS-FD: 40 mg/day) or ideal body weight (IBW)-based adjusted-dose (NS-AD: 1 mg/kg/day). Twenty one matched non-proteinuric individuals (C-FD) also received fixed-dose. Co-primary outcomes were: the achievement of low- and high-VTE risk threshold of antifactor-Xa activity (anti-FXa) defined as 0.2 IU/mL and 0.3 IU/mL, respectively. Low-VTE-risk threshold was achieved less often in NS-FD than C-FD group (91 vs. 62%, *p* = 0.024), while the high-VTE-risk threshold more often in NS-AD than in NS-FD group (90 vs. 38%, *p* < 0.001). Two VTE were observed in NS during 12 months of follow-up (incidence: 5.88%/year). In both cases anti-FXa were 0.3 IU/mL implying the use of anti-FXa >0.3 IU/mL as a target for dose-adjustment logistic regression models. We determined the optimal dose/IBW cut-off value at 0.8 mg/kg and further developed bivariate model (termed the DoAT model) including dose/IBW and antithrombin activity that improved the diagnostic accuracy (AUC 0.85 ± 0.06 vs. AUC 0.75 ± 0.08). Enoxaparin efficacy is reduced in severe NS and the dose should be adjusted to ideal body weight to achieve target anti-FXa activity.

## 1. Introduction

Nephrotic syndrome (NS) is a rare clinical condition with an annual incidence of 1–3 per 100,000 adults. It may be caused by several histological types of glomerular diseases that damage the filtration barrier, resulting in massive urinary loss of plasma proteins—mainly those of low molecular-weight (<150 kDa), in particular serum albumin (SA, 67 kDa) [1]. The classical definition of NS includes proteinuria of 3.5 g/24 h (or 50 mg/kg/24 h), hypoalbuminemia, dyslipidemia and edema [2]. In nephrotic patients, urinary excretion of small anticoagulant proteins, especially antithrombin (AT, 58 kDa), together with liver overproduction of high molecular-weight coagulation factors, such as fibrinogen, factor V, VIII and von Willebrand factor, leads to the development of hypercoagulable state [3]. Estimated venous thromboembolic events (VTE) rates secondary to NS range from 10–20% (clinically overt episodes) to 30–40% (overt and subclinical cases) [4,5,6,7,8]. The risk increases in parallel with NS severity measured by the degree of hypoalbuminemia, and is particularly high for SA ≤ 2.5 g/dL. In such cases, prophylactic anticoagulants use is recommended [2,5,6].

Patients hospitalized for a new episode of NS typically present multiple factors besides hypercoagulability that increase the risk of VTE, including: edema and impaired venous peripheral blood flow, prolonged bed rest, use of high doses of diuretics including continuous intravenous infusion of loop diuretics, and high doses of corticosteroids [2]. In this light, efficient VTE prophylaxis is a crucial safety issue. However, data on indications for prophylaxis, optimal drug, duration of treatment and its effectiveness are very limited and of low quality [9]. Although the current guidelines indicate vitamin K antagonists (VKAs) as preferred drugs for long-term VTE prophylaxis in NS [2], low molecular-weight heparins (LMWHs) remain the first-line anticoagulants used during inpatient treatment.

LMWHs are made up of polysaccharide chains of 4–6 kDa containing a unique pentasaccharide sequence that binds and activates AT. The AT-LMWH complex inhibits the final common pathway of the clotting cascade by 1000-fold enhancement of inhibition of active factor X (Xa), and, to a much lesser extent, of thrombin (IIa). The wide use of LMWHs is related to their favorable pharmacokinetics and pharmacodynamics, including bioavailability of nearly 100%, volume of distribution limited to intravascular space, no significant binding to plasma proteins, endothelial cells and macrophages, no hepatic metabolism and relatively slow renal clearance, resulted in half-life of 3–4 h [10]. Consequently, LMWHs are considered safe drugs with predictable anticoagulant effect that is only dependent on dose, patient’s body weight (BW) and renal function.

Monitoring of antifactor-Xa (anti-FXa), a laboratory marker of LMWH activity, is recommended only in certain clinical situations when drug effect is less predictable, including advanced renal insufficiency with estimated glomerular filtration rate (eGFR) lower than 30 mL/min/1.73 m^2^, extreme obesity, underweight or pregnancy [11]. Although it has been reported that LMWH efficacy may be reduced in severe NS [12,13], this hypothesis has not been tested so far and there are no recommendations regarding anti-FXa monitoring in this group. Likewise, the optimal LMWH dose has not been established.

Target anti-FXa for prophylactic LMWHs use in a population at low VTE risk is suggested to be 0.2–0.5 IU/mL, with a standard fixed-dose use, e.g., enoxaparin 40 mg once daily [14]. For high-risk groups (like severe NS), recent studies have proposed to increase the lower threshold to 0.3 IU/mL and to use higher LMWH doses based on BW [15,16,17,18]. Although the safety cut-off point has not been clearly defined, previous data point to anti-FXa above 0.8 IU/mL as associated with increased risk of bleeding [19,20].

Here, we aimed to fill the pertinent gap in clinical knowledge regarding LMWHs efficacy and appropriate dosing in nephrotic patients. To this end we designed the ENOX-inNS study comparing anti-FXa of enoxaparin between nephrotic and non-nephrotic patients, as well as between two dosing regimens in NS. Secondary goals were to evaluate clinical outcomes, identify factors associated with anti-FXa and to determine the optimal dose-adjustment in this group of patients.

## 2. Materials and Methods

### 2.1. Study Design and Participants

The ENOX-inNS was a single-center prospective study. Patients were recruited during hospitalization for the first episode or relapse of NS after at least 1 month of partial remission defined as proteinuria <3.5 g/24 h with SA > 3.0 g/dL. The inclusion criteria were: severe NS of non-diabetic cause defined as nephrotic proteinuria with SA ≤ 2.5 g/dL and age ≥ 18 years.

Subjects who received fixed-dose of enoxaparin for surgical or non-surgical indications were invited to participate in the study as a control group (C-FD) if they had negative urinalysis strip test and no signs of edema. The C-FD group was matched to the NS groups in terms of age, sex, height, weight and renal function.

The exclusion criteria for NS and C-FD groups were: eGFR < 30 mL/min/1.73 m^2^ calculated with simplified Modification of Diet in Renal Disease equation, body mass index (BMI) ≥ 40 kg/m^2^, malnourishment (<45 kg for female, <57 kg for male), present VTE or history of VTE, prior anticoagulation for comorbidities, contraindications for LMWH, pregnancy.

### 2.2. Intervention

C-FD group received fixed-dose of 40 mg of enoxaparin (Clexane, Sanofi Aventis, Paris, France), whereas nephrotic patients were alternately assigned to fixed- (NS-FD group) or adjusted-dose (NS-AD group) of 1 mg per kg of ideal body weight (IBW). IBW was calculated with the following formula: 45.5 + 0.9 × (height − 152.4) for female and 50 + 0.9 × (height − 152.4) for male, with height expressed in centimeters. The estimated dose was rounded down to the nearest 5 mg. Allocation was stratified to SA level (2.0–2.5 g/dL or <2.0 g/dL).

In NS, enoxaparin was given until at least partial remission of NS was achieved. In the C-FD group LMWH was administered as long as clinically recommended, usually up to 14 days.

### 2.3. Data Collection

Demographic, anthropometric, medical history and laboratory data were collected. Laboratory tests included parameters related to renal function (serum creatinine and eGFR), severity of disease (SA, total protein, proteinuria in 24 h urine collection, AT, D-dimer), and overhydration. Overhydration was assessed by bioimpedance spectroscopy technique using Body Composition Monitor device (Fresenius Medical Care, Bad Homburg, Germany). Patients were examined after resting for 2 min in supine position, 4 h postprandial.

After an average of 3 consecutive days of enoxaparin injections, when steady state was achieved, peak anti-FXa was measured. It was determined 4 h post-dose using chromogenic assay (HemosIL Liquid Heparin, Instrumentation Laboratory, A Werfen Company, Bedford, MA, USA). The test measured the amount of exogenous factor Xa inhibited by complex of enoxaparin and patient’s AT, thereby reflecting peak drug activity in vivo.

After hospital discharge, NS groups were followed up for 12 months. The duration of prophylaxis, VTE episodes and severe adverse effects of enoxaparin (bleeding requiring hospital admission and heparin-induced thrombocytopenia) were recorded.

### 2.4. Outcomes

Two co-primary outcomes were established including the achievement of low- and high-VTE risk threshold of anti-FXa. The first endpoint was designed as the percentage of NS-FD and C-FD patients reaching “low-VTE-risk threshold”, defined as anti-FXa of at least 0.2 IU/mL. It was formed evaluate the primary hypothesis of reduced LMWH efficacy in NS (one-tailed), to estimate the final study group size and to stop the study if interim analysis would demonstrate significantly lower efficacy of fixed-dose in NS. The second co-primary endpoint was specified as the percentage of NS-FD and NS-AD patients reaching “high-VTE-risk threshold”, defined as anti-FXa of at least 0.3 IU/mL. It was formed to evaluate the hypothesis of the need for enoxaparin dose-adjustment in NS (one-tailed). Other outcome measures included: number of VTE and drug-related adverse events during 12 months of follow-up.

### 2.5. Sample Size Calculation

We assumed that the 25% lower rate of NS-FD than C-FD patients with target anti-FXa for low VTE risk would indicate the reduced enoxaparin efficacy in NS (one-tailed hypothesis). We calculated that the enrollment of 35 patients per group is required to prove this hypothesis with statistical power of 75% and alpha level of 0.05. Considering the exploratory design of the study, the interim analysis was planned after inclusion of 60% of patients.

### 2.6. Statistical Analysis

Characteristics of the study population was presented using means with standard deviations (SD) or medians with interquartile ranges (IQR: 25–75 percentile) for continuous variables of normal and non-normal distribution (tested with Shapiro-Wilk test) respectively, and using number with percentage for categorical data. Groups were compared using appropriate tests according to the type of variable and data distribution (described in detail in the table and figure legends). Fisher exact test (one-tailed) was used to verify primary hypothesis of reduced LMWH effectiveness (achievement of low-VTE-risk anti-FXa target) and assumption of a need for higher doses in NS (achievement of high-VTE-risk target). Correlations between potential factors and crude or IBW/dose-adjusted anti-FXa were established using Pearson or Spearman rank correlation coefficient according to data distribution. Univariate logistic regression followed by multivariate backward stepwise analysis were performed to determine the optimal models for enoxaparin dosing in severe NS. Models were presented with Receiver Operating Characteristic (ROC) curves, and compared using Hanley’s algorithm [21], whereas Youden’s method was used to ascertain the optimal threshold. The analysis was performed with Statistica 13.1 (Tibco Software Inc., Palo Alto, CA, USA) with *p*-values < 0.05 considered statistically significant.

The study was approved by institutional bioethics committee and registered on ClinicalTrials.gov (Identifier: NCT04558892).

## 3. Results

### 3.1. Study Population

**Figure 1:**
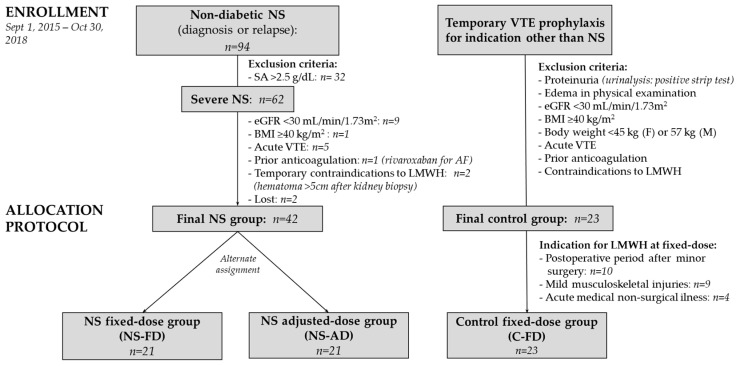
Flowchart of the study protocol. NS, nephrotic syndrome; SA, serum albumin concentration; eGFR, estimated glomerular filtration rate; BMI, body mass index; VTE, venous thromboembolism; AF, atrial fibrillation; LMWH, low-molecular-weight heparin; F, female; M, male; IBW, ideal body weight.

Between 1 October 2015 and 30 November 2018, from a total number of 94 adult nephrotic patients, 62 with severe NS course were identified, and 42 without exclusion criteria were enrolled (Figure 1).

The final study population included 69% male and 31% female, with an average age of 49 years, diagnosed with the first episode (55%) or relapse (45%) of NS. Histological background and severity of NS was similar in both nephrotic groups (Table 1). In comparison to the C-FD group, nephrotic patients presented with lower AT and higher D-dimer levels (Table 1).

Acquired AT deficiency and elevated D-dimer levels were observed more often in severe NS than in the C-FD group (38% vs. 13%, *p* = 0.030, and 88% vs. 45%, *p* = 0.0004, respectively). All of the C-FD patients with increased D-dimers were enrolled in the early postoperative period, 2–3 days after surgery.

### 3.2. Enoxaparin Prophylaxis

Median dose of enoxaparin in NS-AD group was 65 mg (IQR: 50–75 mg). Both fixed-dose groups (NS-FD and C-FD) received 40 mg of enoxaparin, corresponding to 0.51–0.55 mg/kg BW and 0.61–0.63 mg/kg IBW. Concomitant treatment with acetylsalicylic acid was given to 5 nephrotic patients (3 of NS-FD, 2 of NS-AD group) with prior diagnosis of coronary artery disease.

### 3.3. Primary Outcomes

**Figure 2:**
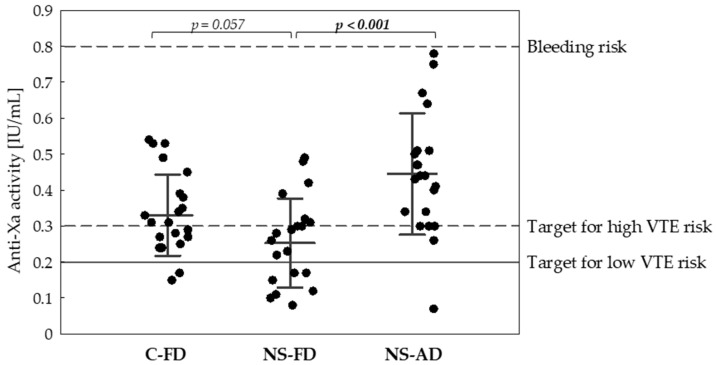
Anti-Xa activity of enoxaparin in the groups. *p*-values of one-tailed Fisher’s exact tests were presented.

Interim analysis showed a reduced enoxaparin efficacy in NS in line with the assumptions made, therefore the study was prematurely stopped. Threshold for low VTE risk (0.2 IU/mL) was reached in 91% of C-FD as opposed to only 62% of NS-FD patients (OR 6.5, 95%CI: 1.2–35.5, *p* = 0.024). According to the second co-primary outcome, threshold for high VTE risk (anti-FXa 0.3 IU/mL) was achieved in 90% of NS-AD patients as opposed to only 38% of NS-FD (OR 15.4, 95%CI: 2.8–84.7, *p* = 0.0005). In general, lower anti-FXa levels were observed in the NS-FD (0.25 ± 0.12 IU/mL) than in the C-FD group treated with the same enoxaparin dose (0.33 ± 0.11 IU/mL, *p* = 0.057), and in the NS-AD group (0.44 ± 0.17 IU/mL, *p* < 0.001) (Figure 2).

### 3.4. Other Outcomes

During 12 months from enrollment, 6 nephrotic patients died due to infections (*n* = 2), malignancies (*n* = 3), and acute coronary syndrome (*n* = 1), while another 2 were lost to follow-up. The remaining 34 patients were followed-up for 1 year. Prophylaxis with enoxaparin was continued for 3 to 52 weeks (median: 3 weeks, IQR: 3–6 weeks, similar in both NS groups). The reasons for drug withdrawal were: remission of NS (*n* = 29, 85%), progression of chronic kidney disease into dialysis (*n* = 3, 9%) or switch to vitamin K antagonists (*n* = 2, 6%). Neither bleeding events nor aFXa values indicative of elevated bleeding risk (>0.8 IU/mL) were observed during enoxaparin administration.

Two males with persistent NS were diagnosed with VTE, corresponding to annual incidence of 5.88% (95%CI: 0.99–14.3). VTE episodes included a case of pulmonary embolism in NS-AD patient with A amyloidosis and deep vein thrombosis in NS-FD patient with light chains deposition disease with histological pattern of MN. The diagnoses were made at month 8 and 12, respectively. Given that anti-FXa levels were 0.3 IU/mL in both cases, we used the cut-off point of >0.3 IU/mL for further analyses.

### 3.5. Predictors of Enoxaparin Anti-Xa Activity in NS

**Figure 3:**
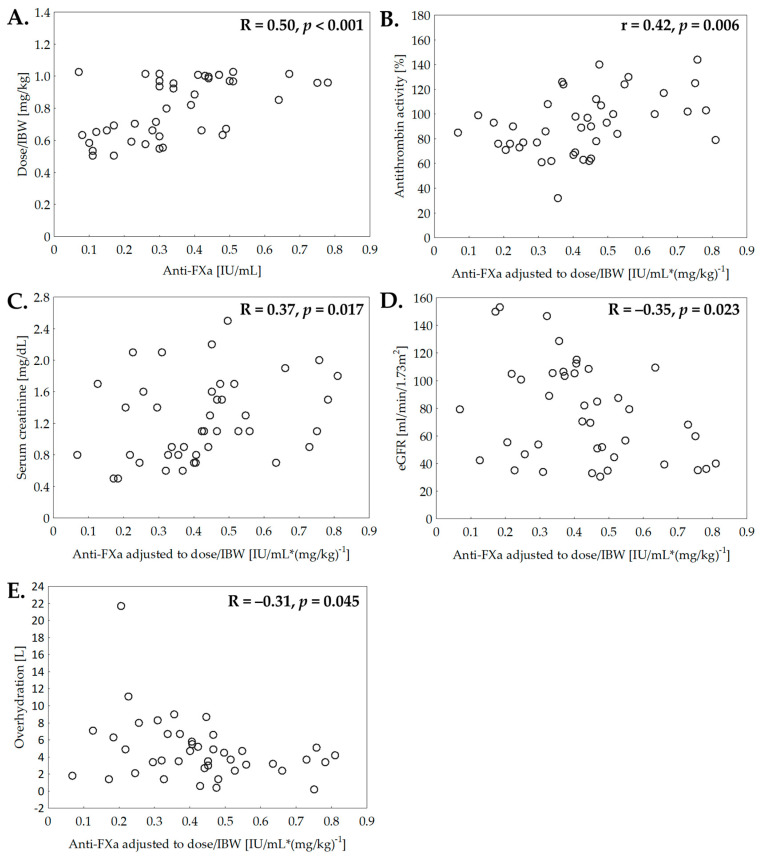
Correlations between enoxaparin concentration and clinical and laboratory m asures in severe nephrotic syndrome: dose/IBW (**A**), antithrombin activity (**B**), serum creatinine (**C**), eGFR (**D**) and overhydration (**E**). R—Spearman rank correlation coefficient, r—Pearson correlation coefficient.

Anti-FXa was strongly correlated with dose/IBW (Figure 3A). Combined analysis of all nephrotic patients revealed the significant relationships between dose/IBW-adjusted anti-FXa and: AT, renal function measures (serum creatinine and eGFR) and overhydration (Figure 3B–E).

**Figure 4:**
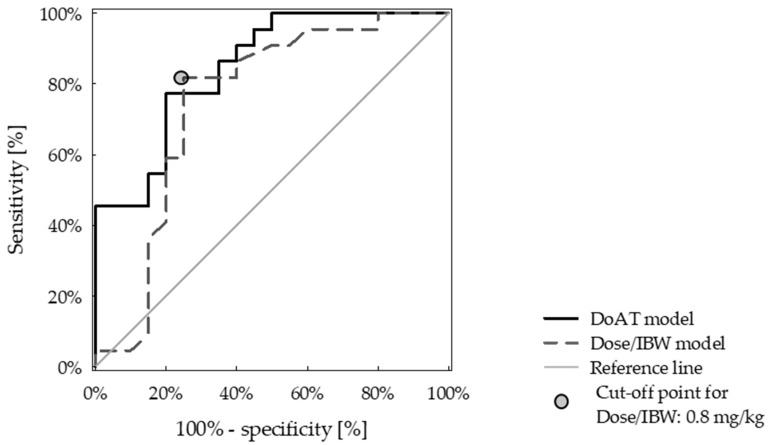
Models predicting anti-Xa activity of enoxaparin in severe nephrotic syndrome.

Among them, only dose/IBW and AT were associated with protective anticoagulation in backward stepwise multivariate logistic regression analysis. A bivariate model based on dose/IBW and AT showed higher predictive value than a univariate model including only dose/IBW (Table 2). However, the difference was not statistically significant (AUC 0.85 ± 0.06 vs. AUC 0.75 ± 0.08, *p* = 0.074) (Figure 4).

The optimal cut-off value of dose/IBW was 0.8 mg/kg, with 82% sensitivity and 75% specificity for identifying patients who will achieve protective anti-FXa levels.

## 4. Discussion

In the present study, we found that the efficacy of enoxaparin is markedly reduced in patients with severe NS compared to patients without proteinuria treated with the same dose, providing a basis for increasing the dose and its adjustment to ideal body weight. Based on drug activity levels in nephrotic patients with VTE episodes, we have redefined a target anti-FXa to >0.3 IU/mL. Target values were achieved by 76% of NS-AD patients as opposed to only 29% of NS-FD. We also identified the optimal dose/IBW cut-off value predicting optimal anti-FXa (0.8 mg/kg), and developed the DoAT model including dose/IBW and AT activity that showed a slightly higher predictive value.

In the study published in 1995, Alhenc-Gelas et al. did not found differences between anti-FXa in nephrotic patients treated with 60 IU/kg of nadroparin (equivalent to enoxaparin dose of 0.6 mg/kg BW) and historical data from general population [22]. However, the study enrolled very small group of nephrotic patients (*n* = 6), with milder disease (SA up to 3.0 g/dL) than our NS group. In another study, Rostoker et al. assessed clinical effectiveness rather than anti-FXa of fixed-dose of enoxaparin in NS [23]. They reported only two VTE episodes among 55 nephrotic patients on prophylaxis, suggesting also that the events may had developed prior to its introduction. This study included milder cases of NS (SA up to 3.0 g/dL) with in-range D-dimer and AT levels. In contrast, we observed an elevated D-dimer and AT deficiency in 88% and 38% of nephrotic patients, respectively. This implies the different risks of VTE in the two studies and makes the comparison of enoxaparin’s clinical effectiveness unreliable.

To the best of our knowledge, no more studies have been published, which makes the evidence for LMWH prophylaxis in NS scarce. Therefore, we conducted the ENOX-inNS study. Our results support the hypothesis of decreased drug activity in this group. The disproportion between anti-FXa in our NS-FD group to non-proteinuric patients is even higher if compared to patients after orthopedic surgery, with acute medical non-surgical conditions or in healthy volunteers (reported anti-FXa ranged from 0.41 to 0.59 IU/mL) than to our C-FD group (mean anti-FXa 0.33 IU/mL) [24,25,26].

Potential mechanisms that may affect LMWH efficacy in severe NS include:

- decreased drug absorption from subcutaneous tissue due to massive edema and reduced peripheral blood flow,
- variable LMWH’s volume distribution due to altered blood volume—according to underfill and overload hypothetic mechanisms of nephrotic edema formation [27,28],
- altered drug pharmacodynamics resulted from acquired AT deficiency due to its urinary loss, and
- increased LMWH’s renal clearance.

We found a significant negative correlation between anti-FXa and overhydration that may indicate lower bioavailability of enoxaparin in NS. As mentioned above, the possible explanation may include malabsorption from the interstitial fluid accumulated in the subcutaneous tissue, exacerbated by reduced peripheral circulation due to altered blood volume. The impact of decreased peripheral blood flow on anti-FXa was confirmed in subjects undergoing vasopressive treatment in intensive care units (ICU) [29]. However, evidence for the link between anti-FXa and edema is sparse, limited to the results of one study that showed no differences of anti-FXa between edematous and non-edematous ICU patients [30]. However, only 7 patients per group were enrolled, anti-FXa was measured after a single-dose instead of assessment at steady state, and patients’ fluid overload was not quantified. In NS, bioimpedance was previously recognized as a reliable tool to assess hydration status [31,32]. Hereby, we used bioimpedance spectroscopy. Our results confirm its value in precise estimation of overhydration in NS and provide additional data on the correlation between anti-FXa and overhydration in nephrotic patients.

The next potential mechanism of reduced LMWH efficacy in severe NS is acquired AT deficiency, which is likely to affect the pharmacodynamics of the drug, since this glycoprotein is a heparin cofactor. We found that AT activity was correlated with anti-FXa, and was a significant factor predicting protective anticoagulation in the DoAT model. Similar relationship has not been observed in ICU patients [33], presumably due to the dominant role of other factors affecting the effectiveness of the drug, such as acute kidney injury or circulatory centralization, as well as the different pathogenesis of AT deficiency [34].

Another issue is the potential link between renal function and half-life of LMWH. In general population, neither dose adjustment nor anti-FXa monitoring is recommended in patients without advanced CKD (eGFR ≥ 30 mL/min/1.73 m^2^), in whom LMWH efficacy is considered independent from renal function [11]. However, it is reasonable to suppose that renal clearance of the drug may be increased in NS due to the damaged glomerular filtration barrier. Furthermore, LMWH may display higher anti-FXa in nephrotic patients even with mildly to moderately reduced eGFR (30–59 mL/min/1.73 m^2^) than in those with normal glomerular filtration (eGFR ≥ 60 mL/min/1.73 m^2^). It is supported by the correlation that we found between anti-FXa and renal function parameters. Several previous studies also shown the relationship between LMWH efficacy and eGFR in non-nephrotic patients without advanced CKD [35,36].

The ENOX-inNS study identified specific NS-related factors affecting enoxaparin efficacy. However, dose/IBW was still the strongest predictor of anti-FXa with correlation coefficient (R = −0.50) comparable to those reported in studies with non-proteinuric patients [18]. We found that the optimal dose/IBW for severe NS was 0.8 mg/kg. In other clinical conditions drug dose was calculated using patient’s current body weight, not IBW [18,37,38]. In NS, however, BW is confounded by fluid overload and shows significant changes during diuretic and immunosuppressive treatment. Therefore, the estimation based on constant parameter, like IBW, seems to be more reliable in this particular group of patients.

The annual incidence of VTE in our nephrotic patients on prophylaxis reached 5.88%. In large retrospective studies significantly lower VTE rates were reported despite the lack of pharmacological prophylaxis, reaching 1.02% patient-year for NS of all-cause and up to 1.7% for MN histology [5,39]. These reports included all cases regardless of severity, while our study was limited to patients with severe course of NS. However, a small study group makes our estimations not precisely stated. Another limitation is the lack of random treatment assignment, despite clinical comparability of both NS groups and demographic and histological features corresponding to the general characteristics of adult NS [40].

## 5. Conclusions

Our findings support the preexisting presumptions about altered pharmacokinetics and pharmacodynamics of LMWH and its decreased efficacy in severe NS. The ENOX-inNS study also highlights the need for dose-adjustment of LMWH or routine monitoring of aFXa in patients with severe nephrotic syndrome. However, further multicenter RCTs are warranted to validate our findings.

## Figures and Tables

**Table 1:** Characteristics of the study population.

Variable	NS-FD *n* = 21	NS-AD *n* = 21	C-FD *n* = 23	*p*-Value		
				NS-FD vs.NS-AD	NS-FD vs.C-FD	NS-AD vs.C-FD
DEMOGRAPHY						
Age (years)	48 ± 20	49 ± 19	49 ± 23	0.995	0.987	0.998
Sex:				0.317	0.717	0.169
Male	13 (62%)	16 (76%)	13 (57%)			
Female	8 (38%)	5 (24%)	10 (43%)			
ANTROPOMETRY						
Height (cm)	168 (165–174)	173.5 (156–179)	170 (160–176)	0.999	0.999	0.999
Body weight (kg)	82 ± 17	80 ± 16	75 ± 14	0.917	0.333	0.565
Ideal body weight (kg)	64 ± 9	64 ± 13	64 ± 11	0.998	0.990	0.978
Overhydration (L)	4.7 (3.1–6.3)	3.6 (2.7–6.6)	0.3 (−0.3–0.6)	0.999	<0.001	<0.001
HISTOLOGY OF NS						
MCD/FSGS	12 (57.1%)	10 (47.5%) ^1^	*NA*	0.780	–	–
MN	5 (23.8%) ^2^	6 (28.6%) ^3^				
AL amyloidosis	1 (4.8%)	1 (4.8%)				
A amyloidosis	0	1 (4.8%)				
Lupus nephritis	1 (4.8%)	0				
No biopsy	2 (9.5%) ^4^	3 (14.3%) ^5^				
LABORATORY TESTS						
Serum albumin (g/dL)	2.1 (1.6–2.4)	2.1 (1.7–2.4)	4.4 (3.8–4.9)	0.999	<0.001	<0.001
Total protein (g/dL)	4.5 (3.9–4.8)	4.5 (4.0–4.6)	7.0 (6.2–7.5)	0.999	<0.001	<0.001
AT activity (%)	96 ± 26	88 ± 23	96 ± 14	0.464	0.991	0.377
AT category:				0.999	0.057	0.057
Decreased (<80%)	8 (38%)	8 (38%)	3 (13%)			
In range (≥80%)	13 (62%)	13 (62%)	20 (87%)			
D-dimer (μg/mL)	0.93 (0.65–1.46)	1.19 (0.87–1.56)	0.36 (0.23–1.09)	0.999	0.030	0.004
D-dimer category:				0.153	0.016	0.0004
Increased (>0.5 μg/mL)	17 (81%)	20 (95%)	10 (45%)			
In range (≤0.5 μg/mL)	4 (19%)	1 (5%)	12 (55%)			
Missed data	0	0	1			
Proteinuria (g/24h)	9.1 ± 4.0	10.9 ± 5.9	*NA*	0.274	–	–
Serum creatinine (mg/dL)	0.9 (0.7–1.5)	1.3 (0.9–1.6)	1.0 (0.8–1.3)	0.534	0.999	0.141
eGFR (mL/min/1.73 m^2^)	79 (52–105)	60 (40–88)	86 (64–105)	0.999	0.999	0.590

AT, antithrombin activity; eGFR, estimated glomerular filtration rate; NA, not applicable. ^1^ including one case of MCD secondary to squamous cell lung carcinoma; ^2^ including one case of MN secondary to lung adenocarcinoma and one case of MN secondary to non-Hodgkin lymphoma; ^3^ including one case of MN pattern due to light chains deposition disease; ^4^ including one case of infection-related glomerulonephritis due to chronic HCV infection; ^5^ including one case of drug-related NS due to sunitinib use. Categorical data was presented as number with percentage and compared with chi-2 test. Continuous variables were described as mean ± standard deviation for normal or median (interquartile range: 25–75 percentile) for non-normal distribution. The comparison of 2 groups were performed with independent samples t-test or Mann-Whitney test, respectively. The comparison of 3 groups using ANOVA with Tuckey post-hoc test or Kruskal-Wallis test with post-hoc test for multiple comparisons, according to the data distribution.

**Table 2:** Logistic regression models predicting protective anticoagulation (anti-FXa > 0.3 IU/mL) in severe nephrotic syndrome.

LogisticModels	Models’ Parameters			Models’ Goodness of Fit	
	Type	β ± SE	OR (95% CI)	AUC (95% CI)	AIC
Univariate	Intecept	−5.02 ± 1.71	–	0.75 (0.59–0.91)	50.54
	Dose/IBW (mg/10 kg)	0.64 ± 0.21	1.89 (1.26–2.84)		
Bivariate(DoAT)	Intercept	−10.53 ± 2.34	–	0.85 (0.73–0.96)	46.20
	Dose/IBW (mg/10 kg)	0.84 ± 0.26	2.33 (1.40–3.88)		
	AT activity (%)	0.04 ± 0.02	1.04 (1.01–1.08)		

IBW, ideal body weight; β, regression coefficient; SE, standard error; OR, odds ratio; CI, confidence interval, AUC, area under the curve; AIC, Akaike Information Criterion. The table summarizes the bivariate regression models that identify patients with protective level of anti-FXa (>0.3 IU/mL). The results of assessment of goodness of fit (AUC and AIC) suggested that DoAT model could be preferred, although the difference between AUCs of models was not significant (see Figure 4).

## Data Availability

The data are available from the corresponding author on request.
